# Determining UAV Flight Trajectory for Target Recognition Using EO/IR and SAR

**DOI:** 10.3390/s20195712

**Published:** 2020-10-08

**Authors:** Wojciech Stecz, Krzysztof Gromada

**Affiliations:** 1 C4ISR Software Department, PIT-RADWAR, 04-051 Warsaw, Poland; Krzysztof.Gromada@pitradwar.com; 2 Faculty of Cybernetics, Military University of Technology, 00-908 Warsaw, Poland

**Keywords:** UAV, trajectory planning, aerial surveillance, vehicle route planning with time windows (VRPTW), electro-optical/infrared camera (EO/IR), synthetic aperture radar (SAR)

## Abstract

The paper presents the concept of planning the optimal trajectory of fixed-wing unmanned aerial vehicle (UAV) of a short-range tactical class, whose task is to recognize a set of ground objects as a part of a reconnaissance mission. Tasks carried out by such systems are mainly associated with an aerial reconnaissance using Electro-Optical/Infrared (EO/IR) systems and Synthetic Aperture Radars (SARs) to support military operations. Execution of a professional reconnaissance of the indicated objects requires determining the UAV flight trajectory in the close neighborhood of the target, in order to collect as much interesting information as possible. The paper describes the algorithm for determining UAV flight trajectories, which is tasked with identifying the indicated objectives using the sensors specified in the order. The presence of UAV threatening objects is taken into account. The task of determining the UAV flight trajectory for recognition of the target is a component of the planning process of the tactical class UAV mission, which is also presented in the article. The problem of determining the optimal UAV trajectory has been decomposed into several subproblems: determining the reconnaissance flight method in the vicinity of the currently recognized target depending on the sensor used and the required parameters of the recognition product (photo, film, or SAR scan), determining the initial possible flight trajectory that takes into account potential UAV threats, and planning detailed flight trajectory considering the parameters of the air platform based on the maneuver planning algorithm designed for tactical class platforms. UAV route planning algorithms with time constraints imposed on the implementation of individual tasks were used to solve the task of determining UAV flight trajectories. The problem was formulated in the form of a Mixed Integer Linear Problem (MILP) model. For determining the flight path in the neighborhood of the target, the optimal control algorithm was also presented in the form of a MILP model. The determined trajectory is then corrected based on the construction algorithm for determining real UAV flight segments based on Dubin curves.

## 1. Introduction

In the literature describing the problems of planning unmanned systems missions, there are theoretical algorithms for determining unmanned aerial vehicle (UAV) flight routes for individual UAVs or UAV swarms that cooperate together. A detailed introduction to the issues of route planning along with the presentation of the taxonomy of the problem is given in the paper by Coutinhoa et al. [1]. Yang et al. [2] present a detailed description of robot 3D path planning algorithms that have been developed in recent years for universally applicable algorithms which can be implemented in aerial robots and ground robots. Depending on the level of route planning details, these algorithms can be divided into general mission performance plans and detailed mission schedules that contain accurate information about flight routes.

The general plans include the allocation of targets for each of the UAVs available during the mission, but they do not specify how to fly to and between destinations. Examples of articles dealing with this type of a problem are given in the paper by Stecz, Gromada [3]. The work in [3] focuses on presenting how to plan fixed-wing UAV missions using SAR during reconnaissance. The concept of mapping flight paths taking into account terrain obstacles was presented. Exact algorithms that can solve this problem were shown in contrast to heuristic methods described in most of papers. On the basis of the presented examples, it has been proved that it is possible to very precisely define the UAV mission plan, taking into account the parameters of its payload.

A specific approach to route planning is a Computational-Intelligence-based (CI) UAV path planning presented in the paper by Zhao et al. [4]. They present different CI algorithms utilized in UAV path planning and the types of environment models, namely, 2D and 3D.

Another type of a route planning problem, where a sensor use is not usually considered, is route planning in real-time. An example is the paper by Schellenberg et al. [5], in which the genetic algorithm implemented on the aerial platform is used to observe volcanic eruptions. The article also introduces the classification of algorithms for unmanned systems, dividing UAVs into Remotely Controlled Systems, Automated Systems, Autonomous Non-Learning Systems, and Autonomous Learning Systems with the ability to modify rules defining their behaviors. Using the classification presented in this work, it can be said that determining optimal UAV flight trajectories for the purpose of target recognition assigns the systems of this type to the Automated Systems’ group.

Detailed schedules determine the exact flight routes along which the UAV should fly, and additionally they take into account the parameters of the sensor assigned to the target [6,7]. This task group includes the recognition planning tasks using a sensor of a specific type for recognition of targets located in a predefined area. There is a wide collection of articles describing tasks in this group, an example of which is the article by Vasquez-Gomez et al. [8].

Preparation of a detailed mission plan for tactical class UAVs was discussed in the article written by Stecz, Gromada [3]. The article presents a way of building a mission plan including a UAV flight, which is to recognize the largest number of the highest priority targets, to which Synthetic Aperture Radar (SAR) has been assigned.

From the optimization theory point of view, when planning a mission based on recognition orders, the global task Vehicle Routing Planning with Time Windows (VRPTW) [9,10,11] is usually solved. The solution of this task guarantees UAV flight between all major targets. A concise overview of exact, heuristic and metaheuristic methods was presented by El-Sherbeny [9]. More advanced models were presented by Schneider [10] and Hu et al. [11]. Schneider [10] presented a variant of the VRPTW that uses specific service times to model the familiarity of the different drivers with the customers to visit. Hu et al. [11] presented how to deal with uncertain travel times. However, the models presented in the articles by Stecz, Gromada [3] and Mufalli et al. [6] did not take into account the way each target was identified, because all available UAV sensors and threats that UAV might encounter during a reconnaissance mission were not taken into account. Detailed guidelines related to the quality of the recognition material that should be obtained were not also taken into account. The quality of the material is determined in the case of EO/IR systems on the The National Imagery Interpretability Rating Scale (NIIRS) (see in [12]), which can be adapted to SAR. From the point of view of optimization principles, therefore, no procedures were presented for solving the local task, i.e., planning the flight trajectory to recognize each of the targets in its location.

This article presents an in-depth analysis of the mission planning method for tactical class fixed-wing UAV and introduces new algorithms related to determining the UAV flight trajectory in the neighborhood of the recognized target. The results of our experience, as part of the construction work on tactical class fixed-wing UAV, are described. The article presents the algorithms for planning the detailed flight trajectory of a fixed-wing UAV, which uses EO/IR and SAR for recognition. As the work concerns tactical class UAVs, the trajectories for the SAR must be particularly carefully defined. Even slight deviations from the designated traffic section may make the prepared SAR scan unreadable. Thus, the task of determining the correct trajectory including terrain obstacles becomes critical. So far, the assumption is that a short-range tactical class system, that operates on a ceiling of 1000–5000 m above the ground level, is considered. By default, its flight length is no more than 6–8 h. This class of systems includes, for example, Hermes 450, which is a tactical class system project.

In the first part of the article, in Section 2.1, the requirements related to the quality of recognition data that should be obtained by UAVs are discussed. In the case of optoelectronic systems, the data quality is described by the NIIRS [12,13]. In the case of SAR, the quality can be expressed in NIIRS or the resolution of the obtained scan [12,13,14]. Principles of flight planning in the neighborhood of the target were discussed when the planner has a terrain map with an altitude grid DTED (for more information about DTED see in [3]). The similarity of the NIIRS to the method of assessing the quality of SAR scans by its resolution was shown.

**Figure 1:**
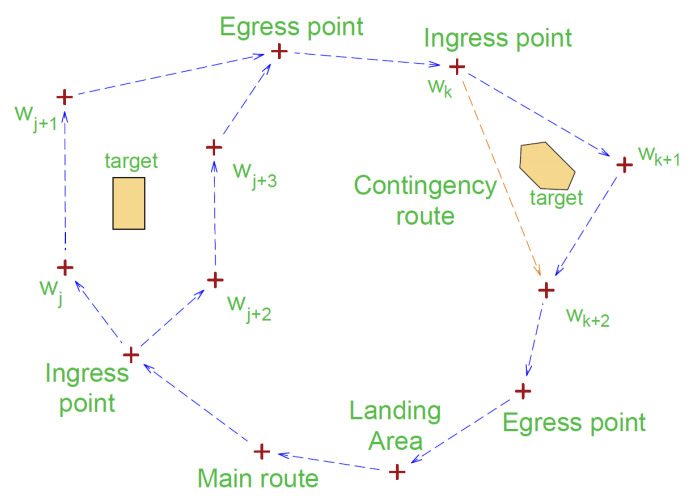
Route plan example with two targets. Orange route segment is an alternative route segment that can be used for target recognition if main route segment should be omitted.

The second part of the article (Section 4) presents an extended model of building a UAV mission plan, which was presented in the work by Stecz, Gromada [3]. The construction of the mission plan, understood as the determination of flight routes between the reconnaissance targets, requires the preparation of a complicated network whose vertices model the UAV flight direction or sensor parameter settings change points, and the network arcs model the route segments on which the reconnaissance is carried out or the route segments of the flight between successive targets. Network construction requires the use of the advanced GIS (Geographic Information System) tools to analyze the area of visibility (related to Line of Sight, see in [3]). In the case of determining the flight path for recognition of a single target, it is assumed that the start and end points of the recognition were determined (so-called ingress and egress points, see Figure 1). The modified model, presented in Section 4.1, takes into account the situation shown in Figure 1, when there is more than one possible UAV flight route in the neighborhood of the recognized target. In the case shown in the figure, the first target can be recognized by UAVs flying one of the route segments $\left(w_{j},w_{j}+1\right)$ or $\left(w_{j+2},w_{j+3}\right)$. The route planning algorithm indicates the preferred route segment, but this can be changed after solving the optimization task (local task presented in Section 4.2).

The third part of the article in Section 4.2 presents the method of determining a detailed UAV flight route in the neighborhood of the target (flight trajectory), based on the general UAV flight plan. The type of a sensor used for recognition is taken into account. Figure 1 shows that flight routes are determined between the entry point (ingress point) to the reconnaissance area and the vertex $w_{j}$ or $w_{j+2}$ and the end point of the reconnaissance $w_{j+1}$ or $w_{j+3}$ and the exit point (egress point) from the recognition area.

When determining the flight trajectory for UAV target recognition, a set of constraints related to battlefield recognition is defined that should be taken into account when planning the flight trajectory. The most important constraints taken into account include the type of a sensor to be used, the quality of the recognition material to be obtained (set on the NIIRS), and the parameters of the UAV itself. In the case of UAV parameters, the most important are operational speed, operational flight altitude, available payload useful for recognition, and time to reach the optimum altitude for the recognition with a given type of a sensor. For these systems, meteorological conditions prevailing in the recognition area (mainly wind speed and cloud cover) are extremely important. This applies also to SAR.

There are several approaches in the literature to solve the local task of UAV flight trajectory optimization. One of them was presented in the work by Stecz, Gromada [3], when the algorithm of modification of the real length of the flight path between points was presented. At that time, it was assumed that the obstacles are convex polygons. For hazards modeled in the form of hemispheres in 3D or circles in 2D, it is better to use dynamic optimization. Models of this class are often converted to MILP models, as shown in the work by Pytlak [15].

For an advanced overview of dynamic optimization rules, see Pytlak [15]. An alternative approach is to use heuristics construction algorithms [16,17,18]. The limitations of these methods are related to the lack of solvers to facilitate the construction and solution of these algorithms.

The rest of the article shows the methods of smoothing the flight trajectory to reflect the real flight trajectory of a given class platform. Due to the size of the platform (tactical class includes UAV up to 150 kg total weight), turning radius and turning time are not negligible. The article presents a new algorithm for the construction of real trajectories of tactical platforms. Based on the definitions introduced by Coutinhoa et al. [1], it can be said that the article presents the problem of planning flight routes and determining the UAV flight trajectory operating alone in which a Dubin’s vehicle model has been used. The flight dynamics is neglected. Multiple waypoints must be visited. The obstacles are present and they are presented as halfspheres. Flight times, velocities, and accelerations are optimization variables.

A novelty of the approach presented in the paper is the integration of the task of global mission planning with local tasks of determining the trajectory of flights between waypoints. In addition, an efficient algorithm for calculation of the actual flight trajectories of the tactical platform having a turning radius exceeding 100 m was presented. All models are MILP models, which guarantee the determination of the optimal solution, if any. In addition, the wide availability of solvers for these tasks allows for the implementation of these algorithms on UAV also for on-line calculation.

Finally, it is worth noting that the UAV system class cannot be detached from the route planning algorithms and the UAV flight trajectory algorithms. In particular, when it is planned to use the sensors of a particular type. Otherwise, simplifying the model makes it useless. In the case of a tactical class system, the most important elements determining the efficiency of its recognition are the operational speed and the maximum flight parameters (speed and height of flight), which are up to 200 [km/h] and 5000 [m] above ground level, respectively.

For each of the presented tasks, exemplary results are provided along with examples of recognition materials collected at the designated trajectories.

## 2. Determining a UAV Flight Route with Payload Usage—Electro-Optical Sensor

### 2.1. Quality of Reconnaissance Products and NIIRS Scale

UAV moving along a route uses a sensor to recognize a predefined target within a range of the sensor. The method of determining the route segments during a reconnaissance, the UAV should follow, depends on a type of a sensor and on the required data quality. In this section the route planning model for a UAV that uses EO/IR and SAR is presented. At the beginning, however, it should be defined how the analyst can select the expected quality of recognition material in both SAR and EO/IR recognition.

NIIRS is a scale used for rating the quality of imagery acquired from various types of imaging systems. It defines the levels of image quality (interpretability) based on the types of tasks an analyst can perform with these images. The higher the NIIRS level, the more details are visible in the image, so the analysts are able to perform more sophisticated interpretations.The NIIRS consists of 10 levels, but taking into account the parameters of UAV, presented in the paper, one can get the images with a scale not higher than 7 (see description of the scale in Bai et al. [12] and NIIRS—National Collection of Aerial Photography [13]). NIIRS is an empirical/subjective scale (the analyst based on his assessment determines the highest level on the scale for which the image meets the requirements). NIIRS level descriptions themselves vary depending on the source of the data.

Examples of some requirements for selected VNIIRS levels (NIIRS for visible light):

- **0**—Image is too low quality to acquire any data.
- **1**—Distinguish between major land use classes (e.g., urban, forest, water, and agricultural). detect a medium-sized port facility; distinguish between runways and taxiways at a large airport [13].
- **7**—Confirm the movement of a car-door, as it is opened or closed, while car is not moving [14].
- **9**—Consistently track the movement of a pedestrian’s body and arms in a busy public area [14].

### 2.2. NIIRS for EO Camera—CIQE Estimation

Optical images are the most popular and best described data collection method, which is why most of mathematical approximations of the NIIRS relate to this type of imaging. The most popular approximation pattern for NIIRS is the General Image-Quality Equation (GIQE).GIQE is an empirical equation used to approximate the NIIRS level of a given image. Currently, the version widely used is GIQE 4, developed in 1996. It considers both raw images and images with processing performed to improve their quality (e.g., sharpening edges). Processing operation parameters must be constant and known. The definition of GIQE 4 is presented in Equation (1).

The newest version of GIQE (version 5) is similar, but it uses less parameters and does not consider image processing. GIQE 5 is not considered in this paper due to the lack of support for IR imaging.

(1) $$NIIRS=ST+A\log_{10}\left(GSD\right)+B\log_{10}\left(RER\right)-0.344\frac{G}{SNR}-0.656H$$

where parameters are the described below.

**Figure 2:**
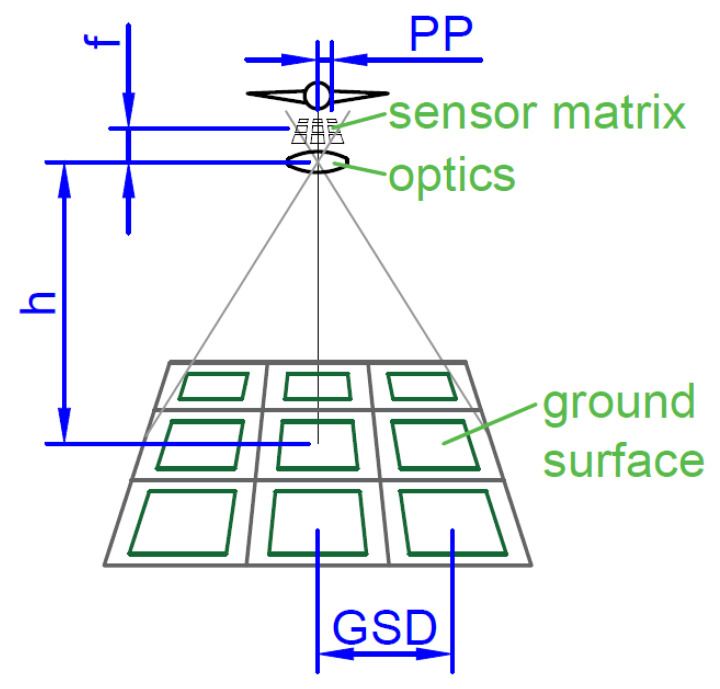
An example of projecting the matrix on the earth surface at a right angle—Ground Sampled Distance (GSD) definition.

- **ST (Sensor Type)**—the value determined by the sensor type is 10.25 for EO and 10.75 for IR.
- **GSD (Ground Sampled Distance)**—field distance corresponding to the distance between two adjacent image pixels (expressed in inches) (see Figure 2).
  GSD in the GIQE equation is determined as a Ground Plane projection. In the case of orthogonal projection, the formula for GSD is as follows,
  (2) $$GSD_{⊥}=\frac{p_{p}}{f}h\;,$$
  where $p_{p}$—matrix pixel pitch, *f*—focal length, and *h*—flight height above ground level.
  For high viewing angles (close to 90°), one can approximate this value with Equation (3):
  (3) $$GSD_{GP}=\frac{GSD_{⊥}}{sin(\epsilon )}\;,$$
  where ϵ denotes look angle (90° for perpendicular)—an angle between an observed earth surface and a flat plane with a normal vector lying on a line connecting a UAV camera with a globe center. At lower values, GSD longitudinal (Along Scan) and transversal (Cross Scan) should be averaged:
  (4) $$GSD_{Along}=\frac{GSD_{angled}}{sin(\epsilon +\alpha -90{}^{\circ})}=\frac{\frac{p_{p}}{f}\cdot SlantRange}{-cos(\epsilon +\alpha )}=\frac{\frac{p_{p}}{f}\cdot \frac{h}{cos(\alpha )}}{-cos(\epsilon +\alpha )}\;\;,$$
  where α—camera look down angle (90° for vertical).
  $GSD_{Cross}$ can be calculated with the similar equation as $GSD_{GP}$, but taking into consideration both angles (5):
  (5) $$GSD_{Cross}=\frac{GSD_{⊥}}{sin(\epsilon +\alpha -90{}^{\circ})}\;,$$
- **Relative Edge Response (RER)**—A fixed value that indicates the camera’s ability to image sharp edges. This parameter is usually determined empirically based on the observation of a known test pattern. Two more coefficients in Equation (1) depend on RER—*A* and *B*, where
  $$A=\left\{\begin{array}{c} -3.32\;for\;RER\geq 0.9\\ -3.16\;for\;RER<0.9 \end{array}\right.\;,$$
  $$B=\left\{\begin{array}{c} 1.559\;for\;RER\geq 0.9\\ 2.817\;for\;RER<0.9 \end{array}\right.\;,$$
   
- **Signal-to-Noise Ratio (SNR)**—The signal-to-noise ratio of the matrix can be determined experimentally or on the basis of formulas taking into account the detailed parameters of the matrix.
  (6) $$SNR=\frac{\mu }{\sigma },$$
  where μ is average pixel intensity and σ is standard deviation of pixel intensity.
  SNR results from photon noise, dark current, FPN (Fixed Pattern Noise), and reading noise. However, it is often also given in sensors datasheets.
  In the paper byVelenzuela, Reyes [19], the authors warn that in GIQE 4, the SNR means precisely Signal Difference to Noise Ratio (SDNR) not SNR.
- **G (Noise Gain)**—A parameter resulting from the operation of edge sharpening, which increases the effective RER, but also increases the noise in the image. For matrix sharpening filters, the gain is determined as the root of the sum of squares of the individual values of the filter mask. An example considering a typical edge detecting filter matrix (7) is shown in Equation (8):
  (7) $$M_{filter[3x3]}=\left[\begin{array}{ccc} 0 & -1 & 0\\ -1 & 5 & -1\\ 0 & -1 & 0 \end{array}\right]\;.$$
  Gain equals:
  (8) $$G=\sqrt{4\cdot {\left(-1\right)}^{2}+5^{2}}=\sqrt{29}\approx 5.4\;.$$
  For images without edge sharpening performed G = 1.
- **H (Height of Overshoot)**—value of overshoot of intensity resulting from edge sharpening. Due to that operation, MTFC (Modulation Transfer Function Compensation) is introduced. It causes additional distortions in the image resulting from the amplitude overshoot.

The parameters RER, H, and G are defined after image postprocessing, by contrast SNR is calculated for an unprocessed image. GIQE applies to uncompressed images. In case of the usage of a compression additional loss of NIIRS has to be assessed for the used algorithm with its degree of compression.

Based on the presented equation, it is possible to define technical (semi-objective) requirements for the given optical or SAR image required to provide sufficient quality. The procedure for determining maximum Ground Sample Distance for given NIIRS value is presented in Algorithm 1. A calculated value is approximated maximum GSD image to maintain the demanded NIIRS level.
**Algorithm 1** Reverse NIIRS calculation—requirements of flight geometry to achieve given NIIRS1:Input expected NIIRS,2:Calculate G, H values for currently used video processing filters,3:Read constant NSR, RER, A and B coefficients,4:$A\log_{10}\left(GSD\right)=NIIRS-ST-B\log_{10}\left(RER\right)+0.344\frac{G}{SNR}+0.656H$  5:$GSD\leq 10^{\frac{NIIRS-ST-B\log_{10}\left(RER\right)+0.344\frac{G}{SNR}+0.656H}{A}}$  (*A* < 0)

### 2.3. Sensor Field of View Calculation

When designing the flight path associated with observing a specific area, the Field of View (FOV) of the selected sensor must be specified. This information is also useful for recordings playbacks. It is worth noting that the calculations estimating the FOV depend not only on the sensor type, parameters, but also on the method of its installation and manipulation on the platform.

An example of a typical camera with a manipulator with two rotary axes will be discussed: C (around Z axis) and A (around X axis, affected by first axis). This implies that the upper and lower edges of the image will be marked by horizontal lines in the camera projected on a plane.

**Figure 3:**
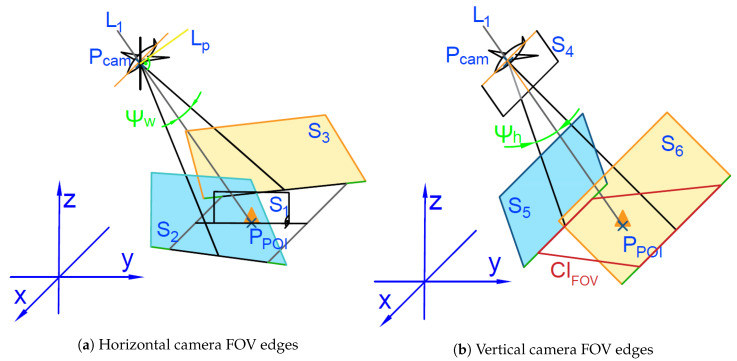
Graphical representation of geometries used in algorithm and its output: $Cl_{FOV}$ closed loop defining outer edges of Field of View.

The method of determining the image border is described in the Algorithm 2, and presented in Figure 3a for a vertical edges calculation and in Figure 3b for a horizontal one. No mathematical equations are described due to many possible representations of each geometry and variety of calculation methods (especially in case of digital maps usage).
**Algorithm 2** Field of Vision calculation (based on Figure 3)1:Indicate camera angles of view based on achievable focal lengths and required resolution/NIIRS,2:Find vertical surface $S_{1}$ going through point of interest $P_{POI}$ and camera $P_{cam}$,3:Find 2 surfaces $S_{2},\;S_{3}$ crossing $S_{1}$ under half width angle of view $\Psi_{w}$ on the semi-vertical line $L_{p}$ perpendicular to line $L_{1}\left(P_{POI},P_{cam}\right)$ and going through $P_{cam}$,4:Find surface $S_{4}$ lying on $L_{1}$ and horizontal line perpendicular to it,5:Find 2 surfaces $S_{5},\;S_{6}$ crossing $S_{4}$ under half height angle of view $\Psi_{h}$ on the horizontal perpendicular to line $L_{1}$,6:Find closed loop indicating edges of FOV-$Cl_{FOV}$ on the surface of the earth model (as flat surface, sphere or digital map) where it crosses with surfaces $S_{2},\;S_{3},\;S_{5},\;S_{6}$; $Cl_{FOV}$ precisely indicates area seen by camera lens.

It is worth adding that with such a configuration, an identical algorithm can be considered for SAR. In the case of a fixed installation, the viewing angles are fixed (affected only by UAV orientation). SARs generally have much larger scanning angles than optical cameras, and therefore the algorithm accuracy will be much more influential in their case.

## 3. Determining a UAV Flight Route with Payload Usage—Synthetic Aperture Radar

### 3.1. NIIRS for SAR

A very detailed introduction to remote sensing can be found in the paper by Colomina, Molina [20]. The authors presented the recent UAV, sensing, navigation, orientation and data processing developments for UAS photogrammetry and remote sensing. They concentrated on the nano-micro-mini UAS segment.

In case of SAR, an image resolution is defined by digital parameters set before data acquisition. Short description of these systems can be found in Gromek et al. [21]. For example resolution of 30 cm means that each pixel will represent an area of 30 × 30 cm regardless of a distance to an object. The higher quality and the slant range, the longer the scan distance (aperture length) must be, as shown in Equation (17) [3]. Factors, that cannot be controlled during the mission or can be affected only indirectly are: acquisition and processing error, external signal disturbances, atmospheric conditions, and aberrations of straightness of the UAV flight.

Analog/digital converters precision or digital processing noises can be treated as a constant factor reducing the NIIRS level. In case of external disturbances, it is difficult to predict and consider their influence on image quality. Atmospheric conditions have little direct influence over the SAR signal, but they affect the UAV flight, thus the closer the flight is to the scanned area (the shorter the path is) the lesser is the weathers influence.

NIIRS applies for SAR images as well as for EO images. Due to a difference in the characteristics of sensors, different requirements are set for each of them to be accounted to the respective NIIRS level (e.g., SAR cannot detect plastic objects). In case of detectable objects, interesting for military and security services (vehicles and buildings), the differences are insignificant.

The SARs beam angles spread wider than a size of an output image. Due to the higher signal power density, radar should be imaging an area as close to the center of the beam as possible. Nonetheless in appliances, where movement limitations can apply (due to threat zones or optimization of a flight trajectory), algorithms should include the options related to scanning with a squint angle.

There is no single, most popular GIQE equivalent for SAR/NIIRS estimations. One algorithm (presented in Equation (9)) was proposed in Gutchess et al. [22].

(9) $$NIIRS_{SAR}=5.606-0.763R+3.657\alpha -3.422\alpha^{2}\;,$$

where *R* is yjr slant range and α is the look down angle (depression angle).

Another alternative approximation was introduced in Nolan et al. [23]. Output equation, shown in (10), has similar form as GIQE, but its coefficients depend on an elevation angle according to a simple table. For example, for the elevation angle 15°: a=5.29, b=−1.91, c=0.32.

(10) $$NIIRS_{P}=a-b\cdot log_{10}\left(GSD\right)+c\cdot log_{10}\left(log_{10}\left(1+SNR\right)\right)$$

In case of SARs, there is a major problem in creation of an universal equation due to many parameters affecting image quality including algorithms and the used principle of data processing. Thus, NIIRS should be researched for every device.

### 3.2. SAR Scans from a Variable Angle

For applications, where tactical UAVs are used, it is important to enable a scanning area under the angle from a perpendicular (default) direction. In military appliances, it might be related to speed flight optimization, but it also needs to avoid threat zones. In case of civil applications, like border guarding, it is convenient to gather scans of few separate areas with a single flight segment. Especially while monitoring the whole space with the Moving Target Indicator (MTI) [24], which might detect POIs in different spots.

**Figure 4:**
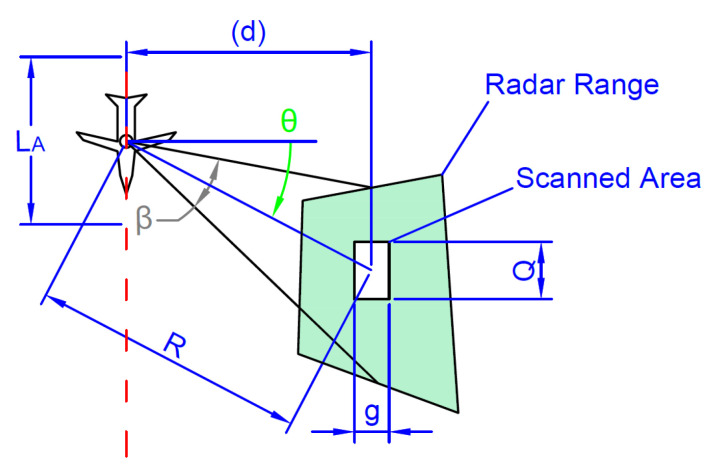
Dimensions sketch in horizontal plane for single scan with SAR under the squint angle θ.

An example geometry with the squint angle (θ≥0) is shown in Figure 4. Moreover, in case of motionless montage of sensor it is required to precisely define geometrical dependencies between the angles.

**Figure 5:**
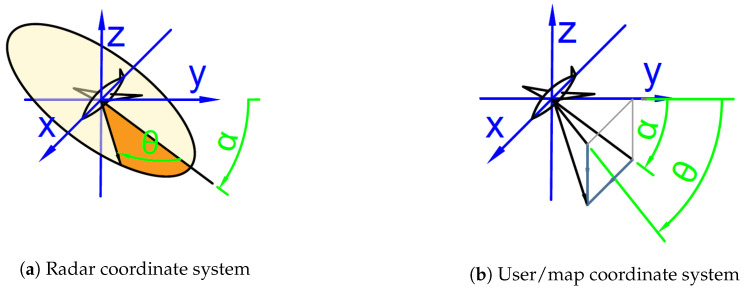
Coordinate systems used in mission planning and execution.

The described radar is mounted under the constant angle (α) in the axis A (around X), the squint (θ) allows to swipe around in the axis C (rotation around Z) which is affected by previous rotation. The angle θ describes a vector lying on a plane defined by the axis X and a normal vector for radars’ antenna (Figure 5a).

In this coordinate system, θ=90° angle implies flight on the level of the ground/object height. For an operator or a program planning flight route, it is better to use a spherical coordination system (Figure 5b).

To change coordinate systems, the most universally used method is the use of translation matrices. Given matrices allow for axis displacement correction for both rotation mechanisms, but in a real application these are negligible due to the fact that a slant range to an object is several orders of magnitude larger than any of the displacements.

The algorithm uses the unit vector e=[0,1,0] to define the vector of observation (lens center axis). The radar coordinate system calculation is introduced in Equation (11).

(11) $$\vec{w}=T_{\alpha }\times T_{\theta }\times \vec{e}\;,$$

where rotation matrices used are defined as Equation (12).

(12) $$T_{\alpha }=\left[\begin{array}{cccc} 1 & 0 & 0 & \Delta x_{\alpha }\\ 0 & cos(\alpha ) & -sin(\alpha ) & \Delta y_{\alpha }\\ 0 & sin(\alpha ) & cos(\alpha ) & \Delta z_{\alpha }\\ 0 & 0 & 0 & 1 \end{array}\right]\;T_{\theta }=\left[\begin{array}{cccc} cos(\theta ) & -sin(\theta ) & 0 & \Delta x_{\theta }\\ sin(\theta ) & cos(\theta ) & 0 & \Delta y_{\theta }\\ 0 & 0 & 1 & \Delta z_{\theta }\\ 0 & 0 & 0 & 1 \end{array}\right]$$

For an optical camera coordinate system, a calculation is presented in Equation (13).

(13) $$\vec{w}=T_{\theta }\times T_{\alpha }\times \vec{e}$$

The presented algorithm enables to calculate what a sensor could see under the given angles. Often it is required to determine where (under what angles) the sensor should scan to view an area of interest. This requires a solution of inverse kinematics, which outputs an angular position for servomechanisms or electronic correction systems for SARs. Simplified (omitting displacements) equations for SAR coordinate reversion are presented in Equations (14)–(16):

(14) $$\alpha =atan(\frac{z_{\vec{w}}}{y_{\vec{w}}})\;,$$

(15) $$\vec{w}=T_{\alpha }\times T_{\theta }\times \vec{e}\Leftrightarrow T_{\alpha }^{-1}\times \vec{w}=\vec{w_{XY}}\;,$$

(16) $$\theta =atan(\frac{y_{\vec{w_{XZ}}}}{x_{\vec{w_{XZ}}}})\;,$$

First, the angle α has to be calculated (14). Based on this angle, the reverse matrix can be used to project $\vec{w}$ on the XY plane (presented in Equation (15)). This leaves simple 2D vector to read θ angle as shown in Equation (16).

### 3.3. SAR Continuous Scan Planning

SARs enable to conduct single scans or series of scans. A sequential scan can be conducted as a single long, continuously expanded strip scan or as a mosaic pattern of separate scans, depending on the radars’ processing software. In case of mosaic output images, depending on the used algorithms and parameters, it can overlay or not. A minimal distance of flight to execute a single scan is determined by the required synthetic aperture length ($L_{A}$) defined in Equation (17).

(17) $$L_{A}=\gamma R\lambda /2\delta_{cr}\;,$$

where γ is the constant multiplier for the given radar, *R* is the slant range, λ is the electromagnetic wavelength, and $\delta_{cr}$ is the expected resolution. For more information see Stecz, Gromada [3].

In case of mosaic scan, the lengths of flight should be the aperture length $L_{A}$ (Equation (17)) multiplied by a natural coefficient as presented in (18). In case of continuous scan, it can be any length greater than the minimal $L_{A}$:

(18) $$L_{flight}=L_{A}\cdot n=\gamma R\lambda /2\delta_{cr}\cdot n\;,$$

where *n* is the coefficient depending on requirements of a size of the image (n∈N).

**Figure 6:**
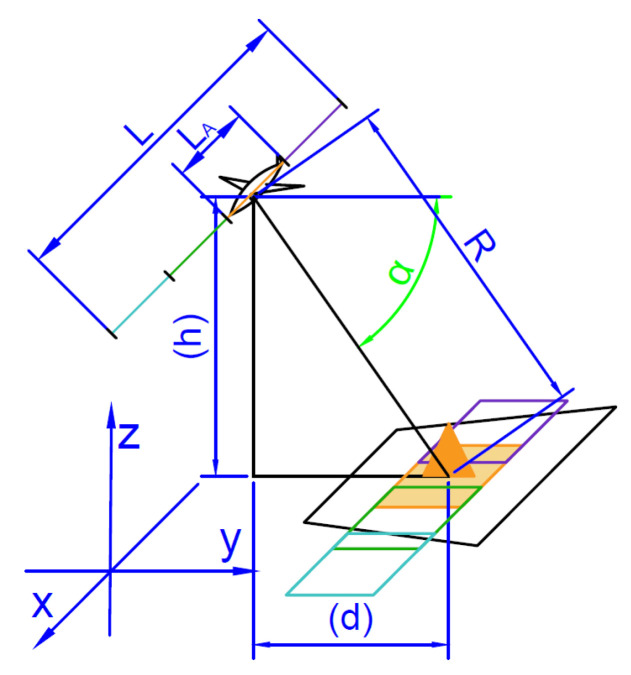
Generated parameters by algorithm.

In case, where the created scan image is longer than the aperture length ($Q>L_{A}$), the created mosaic is overlapping (shown in Figure 6). In the opposite case ($Q<L_{A}$), the output image is not continuous.

A slant range does not change, as the radar is scanning the area constantly in the same direction, which means constant or close to constant distance to the scanned area (when flying in the straight line R≈const).

## 4. UAV Flight Planning Procedure Using Sensors to Recognize Targets

**Figure 7:**
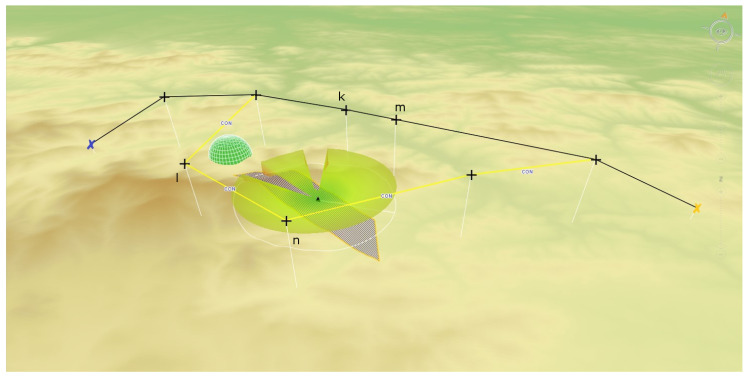
Route plan for UAV with predefined target that is to be recognized with SAR. The green semi sphere models the location of anti-aircraft defense. Dark blue polygon models the Field of View of SAR. Target to be recognized is placed in the middle of the polygon and is marked by a dark triangle.

For the purpose of solving the route planning problem, the network S=<V,E> is introduced that models the area of UAV activity. The vertices V model the waypoints of the network S, the arcs E model the route segments that can be used by UAV when flying from the point number i∈V to the point number j∈V where (i,j)∈E. The reference network model for route modeling is shown in STANAG 4586 ([25]). The targets are near some network arcs what was presented in Section 2.1. Due to their recognition capabilities, tactical UAVs do not need to fly directly above the recognized target. Figure 7 shows an example network.

The presented mission planning problem is to find the routes for UAV, that minimizes flight time and/or ensures recognition of the most important targets. Many constraints affecting the implementation of the mission are taken into account. For example: the UAV parameters like maximum range, the presence of threats, the required time windows in which UAV should reach their destination. These constraints are included in the model presented in the paper in the form of a Mixed Integer Linear Problem (MILP) task in Section 4.1.

The presented procedure for preparing the mission plan has three main steps and it is presented in Algorithm 3.
**Algorithm 3** Route planning algorithm1:Import data: Ψ (set of targets), O (set of danger zones), F (set of airfields)2:Select the route segments assigned to all targets ψ∈Ψ and sensors ω∈Ω used to recognise target (Section 2.1)3:Find feasible route plan for UAV between airfields i,j∈F⊂V on S4:Update distances among vertices of S using algorithm presented in [3] or algorithm from Section 4.2 using information about obstacles o∈O5:Find optimal route—VRPTW model (19)–(33) (Section 4.1)6:Transform generated path into a feasible trajectories7:  Generate trajectories—MILP model (34)–(46) (Section 4.2.1)8:  Smooth generated trajectories (Section 4.2.2)

At the first step all data is imported. When planning a real mission, obstacles that threaten UAVs must be imported. All targets that UAVs must recognize are also loaded into the model.

Next, for all the targets, the sensors used for recognition of each target are assigned with predefined quality of images that must be collected as described in Section 2.1. For each target, which will be recognized using a given type of sensor, the UAV flight segment modeled by arc (i,j)∈E of S is chosen. The UAV flight segment in the neighborhood of the recognized target must be located at the optimal distance for reconnaissance. According to Section 2.1, for each arc (i,j)∈E, describing the flight segment near the target, a set of predefined flight parameters are set.

In the third step all the possible route segments between all pairs of vertices are selected. The vertices model the points lying near the targets to be recognized. For determining the shortest paths between pair of vertices, the Dijkstra algorithm can be used.

Next, at the fourth step, the distances between all pairs of waypoints are updated. The reason is that the network S must be updated with the real travel times for UAV. Therefore, one has to use one of the algorithms described in literature. In the paper by Stecz, Gromada [3], a version of distance calculation for any pair of vertices was presented, where the obstacles were convex polygons. This version is very convenient to use where there is a small number of obstacles according to a number of waypoints. Other way of calculating real flight routes is presented in Section 4.2.1. This approach is based on optimal control problems theory presented in Pytlak [15]. There are some other approaches which use triangulation and Voronoi diagrams ([26]). One of these methods is presented in Xin et al. [18] and Kim [17]. Xin et al. presented a method that plans the paths in 3D space. This method includes two steps: the construction of network and path searching. The construction of network proceeds in three phases and after these phases a net for a path planning is prepared. More complicated version of network construction plausible for path planning is presented by Kim [17]. In this article, multi-robot strategies of terrain exploration is presented in a cooperative manner. The presented strategies do not require global localization of a robot, but each robot builds its own Voronoi diagram as a topological map of the environment.

It is assumed that a robot can communicate. As the sensor network built by one robot meets the network built by another robot, the robots exchange the information with each other. The method presented by Zhang et al. [27] is also worth noting. This paper presents an improved heuristic algorithm based on Sparse $A^{*}$ Search for UAV path planning problem.

As a result of determining the shortest paths between the pairs of vertices, the square matrix $T_{ij}$ is defined, where in any cell of matrix the flight time between the vertices i,j∈V is recorded.

At the fifth step, the routing problem VRPTW is solved. If exists the feasible solution, this solution is also optimal. The details of VRPTW were shown in the paper by Stecz, Gromada [3]. In this paper, one important modification is presented. This additional constraint allow to choose one of alternative route segments assigned to a target, what was shown in Section 2.1. Being able to choose one of the alternative flight paths near your destination is critical to successful mission performance.

In the last step, the optimal trajectories are calculated. This process is divided into two parts. Section 4.2.1 discusses the determination of optimal UAV flight trajectories during reconnaissance of a target between the so-called ingress points and egress points. The presented method allows for determining the optimal flight trajectory between any pair of vertices. Each network vertex has the predefined location ${\left(x,y,z\right)}_{i\in V}$. This is used not only for calculating flight times between destinations but also when the real trajectories are calculated in the second part of an algorithm.

### 4.1. VRPTW Model Redefinition

The section presents the modified version of the VRPTW model for determining UAV flight routes which was originally presented in Stecz, Gromada [3]. The model extensions allow the solver to choose one of the available UAV flight segments during target recognition, which is extremely important in reconnaissance tasks in which UAVs can be destroyed by enemy forces. Figure 1 presents an example of a fragment of the network S on which the optimal flight path is determined. The VRPTW problem is presented in the form of a MILP formulation, so when a solution is found, this solution is feasible and optimal one. The presented model also includes the use of a specific type of sensor for recognition.

Model parameters:

V—set of indices of waypoints (V=1..V) elements.

Each waypoint $w_{i}$ is described by the vector $<{\left(x,y,z\right)}_{i},e_{i},d_{i},p_{i}>,i\in V$, where elements of this vector means: (x,y,z)—waypoint coordinates, *e*—earliest date when an operation or task can start, *d*—date when a planned task should be completed, *p*—priority.

W=V∪F—set of all waypoints and landing bases’ indices.

$T_{WxW}$—travel time matrix, where each element represents a time that UAV needs to fly from i∈W to j∈W.

$T_{1xV}^{ISR}$—ISR matrix where each waypoint has predefined time needed for recognition tasks as a sensor configuration.

Model variables:

$y_{ij}$—1 if UAV travels from i∈W to j∈W; 0 otherwise,

$f_{ij\omega }$—1 if target assigned to the route segment $y_{ij}$ is recognized with the sensor ω∈Ω by any UAV; 0 otherwise

$x_{i}$—1 if UAV travels through the waypoint i∈W,

$t_{i}$—arrival time of UAV to the waypoint i∈W,

An optimization task based on the minimization of the travel time of the UAVs:

(19) $$\rho \sum_{i,j\in W}T_{ij}y_{ij}-\left[\sum_{i\in V}p_{i}x_{i}+\sum_{(i,j)\in E}p_{ij}y_{ij}\right]$$

subject to:

(20) $$\sum_{j\in W}y_{ij}\leq 1\;,\;\forall i\in W$$

(21) $$y_{ij}-1\leq M\cdot f_{ij\omega }\;,\;\forall \omega \in \Omega$$

(22) $$y_{ii}=0\;,\;\forall i\in W$$

(23) $$\sum_{j\in W}y_{0j}=1\;,\;$$

(24) $$\sum_{i\in W:i\neq j}y_{ij}-\sum_{k\in W:k\neq j}y_{jk}=0\;,\;\forall j\in W$$

(25) $$\sum_{j\in V}y_{jb}=1\;,\;\forall b\in F$$

(26) $$t_{j}\geq t_{i}+T_{ij}\cdot y_{ij}-M\left(1-y_{ij}\right)+x_{i}\cdot T_{i}^{ISR},\forall \left(i\in W,j\in W:j\neq i\right)$$

(27) $$x_{i}\cdot e_{i}\leq t_{i}\;,\;\forall i\in W$$

(28) $$x_{i}\cdot d_{i}\geq t_{i}\;,\;\forall i\in W$$

(29) $$x_{i}-x_{j}=0,\forall \left(i\in V:i\in \left\{k,l\right\},j\in V:j\in \left\{m,n\right\}\right)$$

(30) $$\sum_{i\in V:i\in \{k,m\},j\in V:j\in \{k,m\},i\neq j}y_{ij}\leq 1,$$

(31) $$\sum_{i\in V:i\in \{l,n\},j\in V:j\in \{l,n\},i\neq j}y_{ij}\leq 1,$$

(32) $$x_{i}+x_{j}-2y_{ij}\geq 0\;,\;\forall \left(i\in W,j\in W:j\neq i\right)$$

(33) $$\sum_{j\in W:j\neq i}y_{ji}=x_{i}\;,\;\forall i\in V$$

The optimization function is presented in (19). This function has two parts. One part with ρ, as the an optimization coefficient (ρ∈[0,1]) models the travel times of UAVs. Next part models the profits from visiting some number of the targets with the predefined priorities. It is up to the analyst, to prefer the minimization of UAV flight time or prefer the maximization of recognized targets.

Constraint (20) ensures that each target is recognized up to once. It is not necessary to carry out a reconnaissance of the destination if it was not specified in the order [28].

Constraint (21) means that if a target is recognized by UAV, then a proper sensor must be chosen for recognition. This sensor is predefined during a mission plan preparation.

Constraint (22) means that the flights within the same vertex are not allowed. This also applies to landing sites. It should be noted that in the presented model the start and end landing sites always describe a different network vertex of S.

In the article, there is an assumption that every UAV must fly on a mission. This is a technical constraint. In a real situation, there is no need to begin a mission for each UAV. The presented situation is modeled by a constraint (23).

A classical flow preservation requirement for any net is presented as a constraint (24). When the UAV flew into the vertex, it must fly out of the vertex.

Each UAV, that has begun its mission, must return to the landing base b∈W (25).

In the case of the VRPTW problem, additional time window constraints should be added. The time window for recognition is the time it takes for the target to be recognized in order to obtain reliable and useful information. Time window constraints are presented in Equations (26)–(28).

A time of UAV arrival to the waypoint j∈W from the waypoint i∈W is presented in constraint (26). This time must be greater than the time of arrival to the waypoint i∈W plus a travel time between both waypoints and a time needed for reconnaissance task. Constraint (27) prevents UAV from starting the task before the earliest possible date and constraint (28) prevents from starting the task to late.

Figure 7 shows the situation when the planner has identified two possible ways to recognize the target by UAV. In the first case, the UAV can fly after the route segment (k,m)∈E, in the second case, the route segment (l,n)∈E. UAV can start recognition by flying from any vertex k∈V or m∈V (analogously for the second route segment). This maintains the planner flexibility, while planning of target recognition, assuming that the target can be recognized by a UAV flying from several directions. Figure 7 shows this situation for the case of diagnosis using SAR. Constraint (29) forces a solver to choose one of two defined edges to fly through this ((k,m)∈E,(l,n)∈E). Constraints (30) and (31) apply to the requirement to fly over the specified edge. The constraints enforce flight on the indicated edge, but they do not enforce the flight direction.

It should be remembered that the task of VRPTW will be solved correctly if for the indicated network arcs, which have been designated as route segments of the potential UAV flight over the target, the higher priority will be set than for other sections of the route.

Next two constraints are technical constraints that forces solver to choose the proper vertices and edges. Constraint (32) ensures that when UAV travels from i∈W to j∈W then $y_{ijh}$ equals 1. And when $y_{ijh}$ equals 1, then the both waypoints i,j∈W must be visited (constraint (33)). These constraints are obligatory when constraints (29)–(31) are used.

The constraints commonly used to eliminate subtours in VRP were omitted. They were presented in Stecz, Gromada [3] and they are widely known. The first description of them was presented in Miller et al. [29]. Some other simple constraints, like the maximum travel time of UAV, stating that UAV flight time cannot be longer than its maximum possibility were omitted too.

### 4.2. Optimal Trajectory Calculation

The essential part of determining the optimal trajectory for the UAV recognizing the target is to determine the possible trajectory based on the mathematical model presented below. The model takes into account the movement of UAVs in the area where there is probably a threat that is modeled in the form of hemispheres in 3D or circles in 2D. The 2D model is used when it is assumed that the UAV moves at a given height, which is not being changed. These types of threats are most common in practice and they are usually modeled in this way.

The UAV path planning problem is an optimization problem to obtain an optimal cost value under limited constraints, and it is usually modeled as a nonlinear optimal control problem. A good introduction to solving this type of task is the book written by Pytlak [15].

However, some of these tasks can be discretized and solved by classical linear integer methods, as shown in the article in Section 4.2.1. The presented model in the form of a MILP task is used to determine the possible UAV flight path without specifying the exact flight profile. The model allows the analyst to check if the UAV can fly between the inlet point of the recognition area (ingress point) and the start point of recognition. Similarly, the model allows for verification of the possibility of flight between the recognition end point and the exit point from the recognition area (so called egress point), which was described earlier in Section 4 and presented in Figure 1.

As a result of solving the above task, one get two initial UAV flight trajectories. In order to accurately determine the real UAV flight trajectory along predetermined routes, these trajectories should be smoothed, which is presented in Section 4.2.2. Such trajectories show only the actual flight profile of the unmanned platform under optimal conditions, i.e., without taking into account a wind force. However, in order for winds to be taken into account, it is necessary to determine the optimal trajectories for the conditions favorable for carrying out the mission.

#### 4.2.1. Trajectory Calculation

In the described problem, we assume that UAV may encounter obstacles modeled in the form of hemispheres on their flight path. To simplify the description in the article, obstacles are modelled in the form of circles, which means that changes in a flight altitude are not included. This means that the task has a constraint in the form: $|w_{t}-c_{o}|\geq R_{o}$, where t∈N means the number of the next waypoint and o∈O means the index of the terrain obstacle modeled with a circle.

This type of a constraint is a non-linear one and it requires linearization, as described in Parikshit et al. [30] and Lin et al. [31]. To linearize MILP formulation, the non-convex constraint is replaced by a series of linear constraints and binary logical constraints presented in a model formulation (41)–(46).

In the further part of the paper, the waypoints ($w_{t}$) and the obstacles ($c_{o}$) are modeled in 2D. This does not limit the considerations, but it simplifies the form of the model. Therefore, the variable description $w_{ti}$ means the *i*-th coordinate of the point *t*-th and the variable $c_{oi}$ means the *o*-th obstacle center in 2D.

Model variables:

$z_{ti}$—optimization goal of the problem,

$w_{ti}$—UAV position at t∈N modeled by a waypoint,

$u_{ti}$—acceleration at t∈N,

$v_{ti}$—velocity at t∈N,

$b_{tol}$—1 if a UAV position is inside the obstacle *o* for linearization *l*; 0 otherwise,

$g_{ton}$—1 if a constraint is active for linearization *n* and obstacle *o* at time step *t*; 0 otherwise.

The value of $g_{ton}=1$ indicates an active constraint, which is necessary for a correct formulation of the model. For the task of determining the trajectory to be a linear task, the constraint of the form $|w_{t}-c_{o}|\geq R_{o}$ should be replaced by four inequalities, one of which is active in subsequent iterations of the algorithm (see the constraints (41)–(44)).

Optimization task based on the minimization of the travel time of the UAVs:

(34) $$\sum_{i\in D,t\in N}z_{ti}$$

subject to:

(35) $$u_{t,i}\leq z_{ti},\forall \left(i\in D,t\in N:t<N\right)$$

(36) $$u_{t,i}\geq -z_{ti},\forall \left(i\in D,t\in N:t<N\right)$$

(37) $$v_{t+1,i}=v_{ti}+u_{ti}\cdot \epsilon ,\forall \left(i\in D,t\in N:t<N\right)$$

(38) $$w_{t+1,i}=w_{ti}+v_{ti}\cdot \epsilon ,\forall \left(i\in D,t\in N:t<N\right)$$

(39) $$u_{ti}\geq U^{min},u_{ti}\leq U^{max},v_{ti}\geq V^{min},v_{ti}\leq V^{max},\forall \left(i\in D,t\in N\right)$$

(40) $$w_{1,1}=x0,w_{1,2}=y0,w_{T,1}=xN,w_{T,2}=yN,$$

(41) $$\left(c_{o1}-w_{t1}\right)\cdot sin_{l}+\left(c_{o2}-w_{t2}\right)\cdot cos_{l}\geq R_{o}-M\cdot b_{tol}+M\cdot \left(g_{to1}-1\right),\forall \left(t\in N,o\in O,l\in L\right)$$

(42) $$-\left(c_{o1}-w_{t1}\right)\cdot sin_{l}-\left(c_{o2}-w_{t2}\right)\cdot cos_{l}\geq R_{o}-M\cdot b_{tol}+M\cdot \left(g_{to2}-1\right),\forall \left(t\in N,o\in O,l\in L\right)$$

(43) $$\left(c_{o1}-w_{t1}\right)\cdot sin_{l}-\left(c_{o2}-w_{t2}\right)\cdot cos_{l}\geq R_{o}-M\cdot b_{tol}+M\cdot \left(g_{to3}-1\right),\forall \left(t\in N,o\in O,l\in L\right)$$

(44) $$-\left(c_{o1}-w_{t1}\right)\cdot sin_{l}+\left(c_{o2}-w_{t2}\right)\cdot cos_{l}\geq R_{o}-M\cdot b_{tol}+M\cdot \left(g_{to4}-1\right),\forall \left(t\in N,o\in O,l\in L\right)$$

(45) $$\sum_{l\in L}b_{tol}\leq L-1,\forall \left(t\in N,o\in O\right)$$

(46) $$\sum_{n\in 1..4}g_{ton}=1,\forall \left(t\in N,o\in O\right)$$

where: $sin_{l}=sin\left(\left(l-1\right)\cdot \pi /6\right)$ and $cos_{l}=cos\left(\left(l-1\right)\cdot \pi /6\right)$.

Formula (34) is the cost function that considers the control changes only. The calculations also used the quadratic function of the form given in Formula (47):

(47) $$\sum_{t\in N:t<N,i\in D}{\left(w_{t+1,i}-w_{ti}\right)}^{2}\;.$$

Constraints (35)–(36) are linked with an optimization function. They provide that optimization function adds absolute values of control signal values (acceleration). Constraints (37) and (38) are system Equations of the UAV. Constraint (39) ensures that an acceleration and a velocity will not exceed the predefined ranges (they are lower and upper bound constraints of velocity and acceleration). Constraints (40) are initial and finial state boundary constraints. Constraints (41)–(44) are the obstacle avoidance constraints after linearization. Constraint (45) means that for any obstacle and all of the linearizations for any time step if one $b_{tol}=0$, then the trajectory point is outside the circle. And this is preferred situation, i.e., if all the points are outside the circles, then solution is feasible. Constraint (46) guarantees that one of the constraints (41)–(44) will be satisfied. These four constraints are transformation of the absolute value function ($abs[\left(c_{01}-w_{t1}\right)+\left(c_{02}-w_{t2}\right)]$) derived from $|w_{t}-c_{o}|$, so only one $g_{ton}$ can be positive.

#### 4.2.2. Smoothing the Trajectory—UAV Crossing the Waypoint

In order to prepare an efficient path plan, optimal turning approaches must be researched. Every proposed flight order has to be modeled with regard to dynamic limitations of the platform. Otherwise, the quality (cost function) of a path would base on The Euclidean distance. This is very coarse assumption viable only for trajectories, where the turn radius *R* is at least two times smaller than minimal travel distance between two consecutive waypoints. For $R>1/2\cdot min(L_{i})$ realization of a trajectory might require additional maneuvers in narrow turns to reach the required altitude or distance for correct maneuver.

There are analytical algorithms for path planning like Fast Marching (FM [32], which does not consider turning radius and other kinematic boundaries, $FM^{2}$ [32]—creates continuous trajectories, but generated values might exceed UAVs limitations. Thus they can be assigned to path planning algorithms in the areas with dense population of obstacles. Different approach is presented in the paper Janjoš et al. [33] using Quartic Splines an RRTs, but it is defined for a multirotor drone, which carries different dynamic limitation. Adjusting to fixed wing dynamics would affect the quality of the output trajectory.

There are several commonly described algorithms for passing-point maneuvers like: simple past-target turn, which is the simplest one, popular in military aviation, but not intuitive for the planner due to a continual trajectory offset from a line segment directly connecting waypoints; single turn before waypoint (also called “short turn”)—fastest presented by Anderson et al. [34], but does not pass over a waypoint; 3-turn Dubin curve presented by Noonan et al. [35]  and an algorithm introduced by Anderson et al. [34], which is close to a given path and optimal but a calculation is more complex. In the paper, the modified version of 3-arc Dubin algorithm is presented.

It should be noted that the waypoints generated in the process of determining the flight trajectory, show the optimal UAV flight path. Therefore, after calculations, the number of the route points W increases significantly. But we must keep in mind the UAV maneuverability, so in the process of smoothing the flight path, the number of waypoints is minimized. In this section, for the sake of simplicity and without loss of generality, we assume that the waypoint w∈W is described in 2D by the coordinates (x, y).

In the further part, for ease of understanding, an acute or obtuse angle (α<180) side is considered to be the inner side of the turn. For α=180 there is no need for any maneuver.

**Figure 8:**
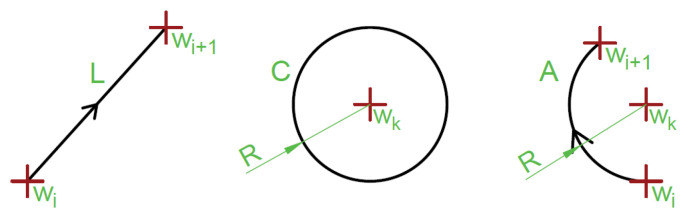
Parameters defining segments, circles and arcs.

In this paragraph, a set of geometry shapes is described. For the higher description precision will be defined using a symbol and an explicit vector of attributes, except for the points (*w*) and the lines (*L*) (as their form depends only on the used metrics). The line segment *L* is defined by a pair of the waypoints $\left(w_{i},w_{i+1}\right)$ referred to as $L(w_{i},w_{i+1})$. The circle C is defined by a pair of waypoints and the radius $\left(w_{k},R\right)$ referred to as $C(w_{k},R)$. The circle arc is defined here as a circle and two waypoints corresponding to the ingress and egress points $\left(w_{i},w_{i+1},C\left(w_{k},R\right)\right)$ referred to as $A(w_{i},w_{i+1},w_{k},R)$. All definitions are shown in Figure 8.

**Figure 9:**
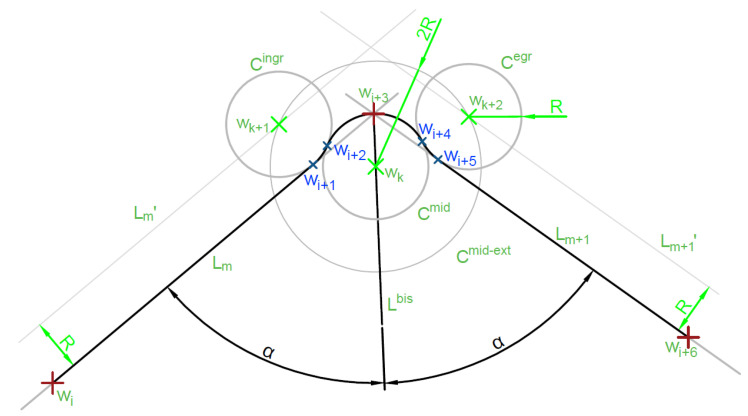
3 arc Dubins curve.

The algorithm that determines how the turn will be executed by the UAV that passes through the waypoint is shown in Algorithm 4. Figure 9 shows graphically how to determine the trajectory in the case of flight through a given waypoint. With this algorithm, the UAV begins turning before the waypoint to minimize the deviation from the optimal flight path.
**Algorithm 4** 3-arc Dubin curve calculation1:Calculate line $L_{m}$ going through target $w_{i+3}$ and ingress point $w_{i}$ also $L_{m+1}$ through $w_{i+3}$ and egress point $w_{i+6}$ (line $L_{m+1}$),2:Calculate 2 lines $L_{m}^{\prime }$, $L_{m+1}^{\prime }$ equations parallel adequately to $L_{m}$ and $L_{m+1}$ offset by *R* to the outside of the turn,3:Find bisector line $L^{bis}$ of angle $∠L_{1}L_{2}$,4:Find point $w_{k}$ on bisector in distance *R* to the inner side of the angle,5:Draw circle $C^{mid}\left(w_{k},R\right)$ and $C^{mid-ext}\left(w_{k},2\cdot R\right)$,6:Find crossing points $w_{k+1}$ and $w_{k+2}$ of $C^{mid-ext}$ with adequately $L_{m}^{\prime }$, $L_{m+1}^{\prime }$,7:Find points of tangency $C^{ingr}\left(w_{k+1},R\right)$ with $L_{m}$ − $w_{i+1}$ and $C^{mid}$ − $w_{i+2}$,8:Find points $w_{i+4}$ and $w_{i+5}$ for $C^{egr}\left(w_{k+2},R\right)$ adequately,9:Draw line segment $L_{m}\left(w_{i},w_{i+1}\right)$, arc $A(w_{i+1},w_{i+2},\left(w_{k+1},R\right))$, arc $A(w_{i+2},w_{i+4},\left(w_{k},R\right))$, arc $A(w_{i+4},w_{i+5},\left(w_{k+2},R\right))$, line segment $L_{m+1}\left(w_{i+5},w_{i+6}\right)$.

Algorithm 4 has the lowest integral quality index among the described algorithms, which is formulated as

(48) $$\int_{w_{i}}^{w_{i+6}}dist\cdot dl\;,$$

where dist is the minimal distance between the calculated trajectory fragment dl and $L_{m},L_{m+1}$ line segments connecting the ingress point with the middle point and the middle point with the egress. Another advantage of this algorithm is its, as previously described, an intuitive trajectory. UAV will be flying on the shortest line segment between two points for most of the time. While the overshoots on the turns will be the lowest from the mentioned maneuver types.

#### 4.2.3. Smoothing the Trajectory—UAV Crossing a Segment

In this algorithm, it is assumed that there was the previous maneuver calculated accordingly to the algorithms described for point (Algorithm 4) and segment targets (Algorithms 5 and 6). This requirement induces the existence of a post-maneuver arc. That allows for creation of a single, universal rule to adjust movement to the next target.

**Figure 10:**
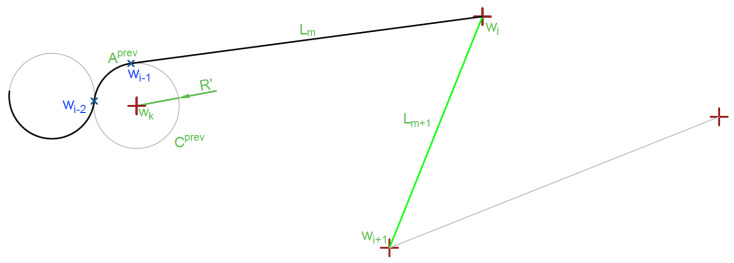
Single arc ingress segment curve calculation—initial state.

**Figure 11:**
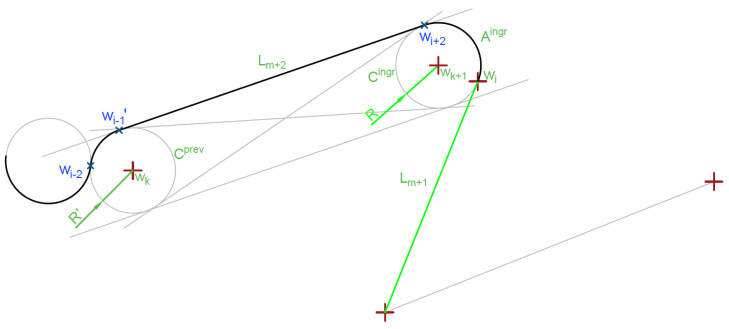
Single arc ingress segment curve calculation result (Algorithm 5).

An algorithm for through-line-segment trajectory generation is described in Algorithm 5 and shown in Figure 10 and Figure 11.

It is important to note that in case of long distances between waypoints, UAV maneuverability and atmospheric conditions can differ considerably. That is why in this algorithm two separate turn radiuses’ symbols are used. *R* is the radius for maneuver with current speed and $R^{\prime }$ is the radius of the last turn taken ($A^{prev}$), usually $R^{\prime }\approx R$.

For this algorithm, also the direction of an arc has to be defined. Direction defines right (clockwise) and left (counterclockwise) turn type using a classical vector Equation (49):

(49) $$dir=\vec{\left(w_{k}w_{i}\right)}\times \vec{\left(w_{k}w_{i+1}\right)}\times {\left[0,0,1\right]}^{T}\;.$$

Turn is clockwise if dir (*z* axis value of vector multiplication) is lower than 0. Turn is counter clockwise for dir>0. dir=0 means $w_{i}=w_{i+1}$ should not occur.
**Algorithm 5** Ingress arc calculation for line segment target1:**if** last maneuver is line segment $L_{m}$ (Figure 10) **then**2:  Delete last maneuver ($L_{m}$),3:Find circle $C^{prev}\left(w_{k},R^{\prime }\right)$ corresponding to previous maneuver-arc turn ($A^{prev}$)4:Find circle ($C^{ingr}$) tangent to the beginning of target line segment ($L_{m+1}$) on the side of previous target (shown in Figure 11),5:**if** distance $|w_{k},w_{k+1}|<\left(R+R^{\prime }\right)$ and $A^{prev}$ direction ≠$A^{ing}$ direction **then**6:  Replace circle ($C^{ingr}$) with tangent to the beginning ($w_{i}$) of target line segment ($L_{m+1}$) on the side further from previous target,7:Find all tangent lines to $C^{ingr}$ and $C^{prev}$,8:Select tangent segment $L_{m+2}$ corresponding to direction of both arcs,9:Adjust previous arc ($A^{prev}$) exit point to $w_{i-1}^{\prime }$,10:Add segment $L_{m+2}$ to maneuver list,11:Add arc $A(w_{i+2},w_{i},w_{k+1},R)$ to maneuver list.

Algorithm 5 introduces offset between the expected target (beginning of the segment $L_{m+1}$) and the real target (tangent point on the circle $C_{ingr})$, that is why the previous arc ($A^{prev}$) must be adjusted and the last segment $L_{m}$ replaced.

**Figure 12:**
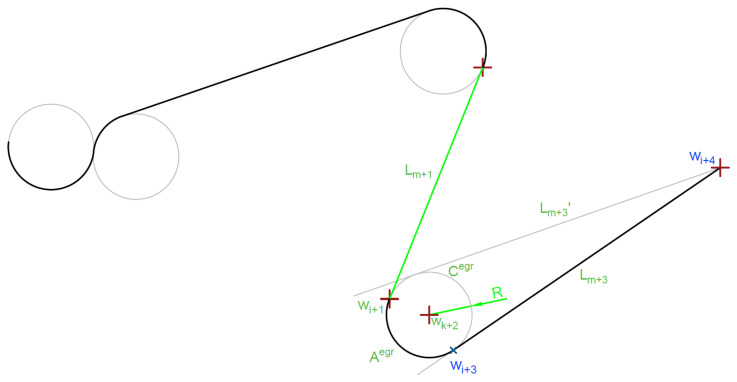
Single arc out segment curve calculation

In ***if*** condition (line 5), an argument distance is compared to $\left(R+R^{\prime }\right)$ not 2·R as previous maneuver could be flown with different parameters—described earlier. This condition checks if there is enough space to execute proper maneuvers connecting both curves. If arc’s directions correspond and $R=R^{\prime }$ (due to a very short distance between arcs, speed cannot change significantly) there will be always 2 tangent segments correctly connecting arcs.
**Algorithm 6** Egress arc calculation for line segment target1:Find circle $C^{egr}\left(w_{k+2},R\right)$) tangent to the end ($w_{i+1}$) of target line segment $L_{m+1}$ on the side of next waypoint $w_{i+4}$ (Figure 12),2:**if**$|w_{k+2},w_{i+4}|<R$**then**3: Replace circle ($C_{egr}\left(w_{k+2},R\right)$) with tangent to the end of target line segment ($L_{m+1}$) on the side further from next target,4:Find 2 tangents ($L_{m+3}$, $L_{m+3}^{\prime }$) to egress circle $C_{egr}$ going through $w_{i+4}$,5:Select tangent segment $L_{m+3}$ co-directional with $C_{egr}$,6:Add arc $A(w_{i+1},w_{i+3},w_{k+2},R)$ to maneuver list,7:Add segment $L_{m+3}$ to maneuver list.

The egress arc Algorithm 6 is simpler because it points at the expected next target, which is precise for the point targets. For the segment target, if needed, will be adjusted as shown in Algorithm 5. If the next mission target requires flight on the line segment $A^{egr}$ and $L_{m+3}$, it will be adjusted in the next step as described in Algorithm 5. The algorithm is presented graphically in Figure 12.

## 5. Results

### 5.1. Mission Plans with Payload Usage

**Figure 13:**
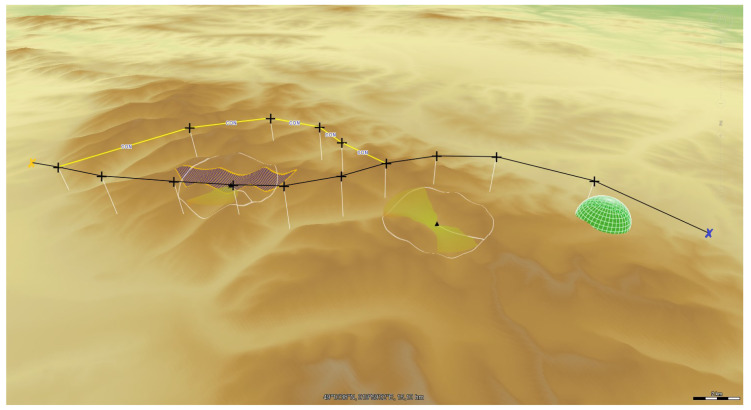
Route plan for UAV with predefined targets that are to be recognized with SAR and EO/IR. Dark blue polygons model the Field of View for SAR and EO/IR, respectively. FoV for EO/IR is usually smaller than FoV of SAR.

The section presents examples of research results related to route planning and determination of UAV flight trajectories. The flight plans were determined in various terrain conditions, from mountainous to lowland. Figure 13 shows examples of recognition targets indicated in the upland area.

The first POI is to be recognized using the SAR, the second using EO/IR. During reconnaissance of the first target with SAR, UAV can fly on one of two possible routes. The algorithm for determining flight routes VRPTW (19)–(33) will choose the safest route in the sense of the given target function.

If the UAV is to recognize a target located in an area where a threat to the UAV is located, a flight trajectory should be determined that minimizes the risk of platform destruction according to the algorithm (34)–(46).

**Figure 14:**
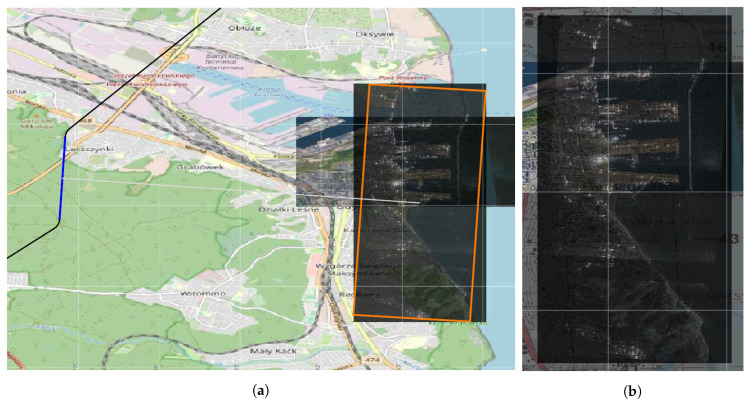
Result of SAR scanning the terrain of port in Gdynia. SAR scan with geographical reference put on map as additional layer. (**a**) Trajectory is presented on the image. Blue segment represents synthetic aperture; (**b**) zoomed SAR scan.

Figure 14a shows the targets indicated for recognition located near the port. The visibility range of the UAV flying at a certain height Line of Sight is presented. Figure shows the flight path determined by the VRPTW algorithm that consists of a few flight segments. Flight trajectory to the area of recognition and the result of the route smoothing algorithm are drawn. Figure 14b shows a SAR scan of the terrain being recognized. The scan has its own geographical reference, so it can be located on the digital map.

**Figure 15:**
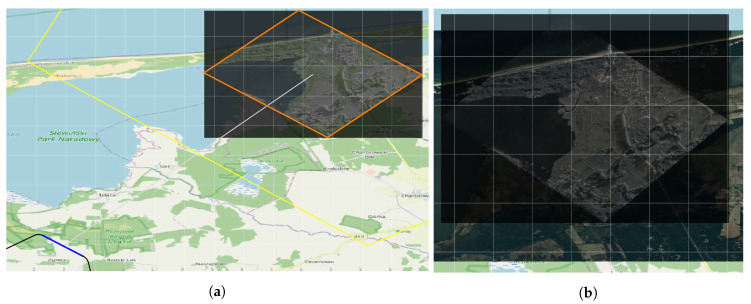
An example of data fusion from EO/IR and SAR. (**a**) The orange polygon shows the scanned area. The area bounded by yellow sections shows Field of View for SAR. Background map is part of OpenStreetMap; (**b**) zoomed SAR scan.

Figure 15 shows another UAV mission plan. UAV recognizes objects near the sea shoreline. UAV uses SAR. Figure 15a shows the route plan with imposed visibility range (LoS) for SAR for each of the indicated targets. The trajectory presented consists of three route segments. The first is the flight segment to the point from which the target recognition begins (ingress point). It is marked in dark. Next, is a flight segment where UAV scans the area. It is marked in blue. The last segment models the flight trajectory from the point where recognition was completed to the exit point from the recognition area (egress point). Figure 15b shows the resulting terrain scan put on the digital map. The scan made by SAR has its own geographical reference.

**Figure 16:**
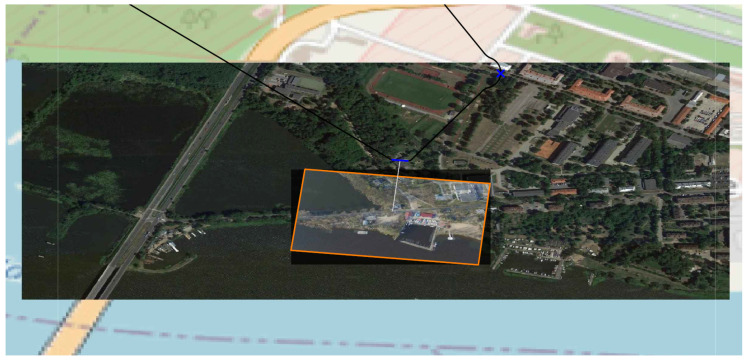
An example of photo data fusion from EO device gathered in different time. UAV trajectory segments are marked. The smaller photo was taken by UAV for recognition of objects located in this smaller area.

The last example presented in Figure 16 shows the photo made by UAV flying over the Zegrze Lake. UAV recognizes objects near the lake. UAV uses only EO head. The trajectory presented consists of three route segments. The photo is put on another photo made earlier which covers larger area. Both photos have got their own geographic references, so it is possible to precisely localize them on the digital map.

The example presented is very often used in practice when the analysts want to check some part of the terrain comparing them with larger photos.

### 5.2. Trajectory Generation and Optimization Function

**Figure 17:**
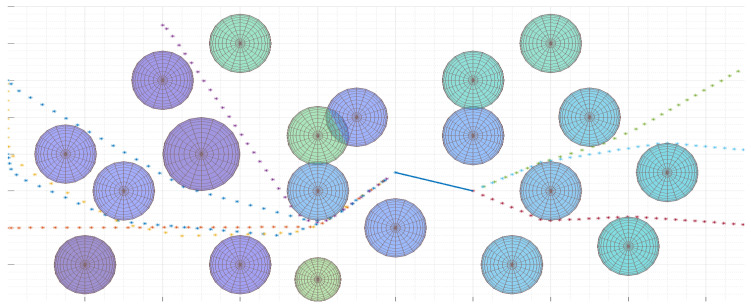
Sample UAV flight trajectories generated for various UAV flight start and end points. The blue line shows the flight segment in which the UAV recognizes a target. Optimization function is defined in (34).

This section discusses the important elements to pay attention to when implementing the UAV flight trajectory generation algorithm. The performance aspects of the algorithm for determining real UAV trajectories are also presented. Figure 17 shows an example area with UAV hazard areas marked. The location of the reconnaissance that UAV will perform is indicated. The place is marked with a line segment. A trajectory, according to Equations (34)–(46), was generated for several randomly selected start and end points.

The goal function was used to minimize controls, see (34). It can be seen that in a few cases the designated routes are not the shortest routes. However, these are routes on which UAVs rarely modify the control values which in practice save energy. Hence, the control minimization function is widely used in solving optimization tasks.

**Figure 18:**
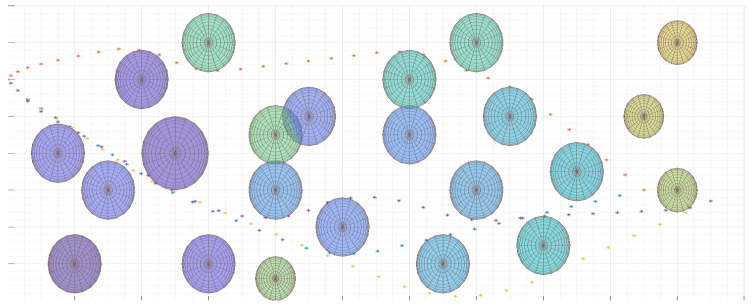
Four UAV flight trajectories generated for different optimization functions. The trajectories for the objective function that minimizes the number of control changes (34) are marked in blue and yellow. The trajectories for the function that minimizes the length of the UAV flight path (47) are marked in red and purple.

Figure 18 shows the final trajectories for two different goal functions. Destinations for two UAVs have been selected. One of them beyond obstacles. The second was indicated in the place between the obstacles. The trajectories described by the objective function, represented by Equation (34), are marked in blue and yellow. It can be seen that these trajectories do not minimize the length of the flight. However, they bypass some obstacles, because the flight between them would force frequent changes of controls. It can be said that the use of the objective function, minimizing the number of steering changes, minimizes the risk of flight in hazardous areas. But, this increases the length of the flight. If it is necessary to minimize the amount of controls, the trajectory determined does not usually coincide with the trajectory determined for the case of minimizing the route length given by optimization function (47). The shown cases indicate that the selection of the target function is of great importance for the design of the flight trajectory. It can be said that the algorithm minimizing the number of control changes avoids UAV flight planning in an area with a large number of obstacles.

It is also worth discussing the conclusions resulting from the analysis of changes in the flight path depending on the optimization function used and the number of obstacles. Many experiments were conducted to check trajectory changes in a situation of changes in the number of threat areas for each of the examined objective functions. It should be noted that the greatest impact on the performance of the flight trajectory generation algorithm (34)–(46) has the number of targets located in the UAV flight area relative to the UAV flight area. Even large numbers of hazard areas at a distance from each other exceeding the radius of the hazard area are not a problem for the algorithm. The problem with determining the trajectory appears when the obstacles are close together. In this case, for 23 obstacles, the determination of the trajectory could exceed 30 s when the place to which the UAV was flying was in the middle of the area (see Figure 18). Otherwise, the trajectories were determined in a few seconds. Due to the fact that the model linearization is used to determine the trajectory, increasing the quality of the mapping of the hazardous area also extends the calculation. The calculations were made using the IBM Cplex environment on a computer with an i3 processor (3.2 GHz).

## 6. Conclusions

The procedure of determining the flight trajectory for a short-range tactical UAV, described in the article, was actually implemented in one of the projects in which the authors participated. The procedure is a part of the construction of the so-called Mission Plan, which consists of a set of routes that UAV can follow during the mission. In the mission plan, methods of using payload used for reconnaissance are also being developed. From the point of view of the optimization task, which is the preparation of the mission plan, in the case of determining the flight trajectory to recognize a target, one can talk about solving the local task. The global task is to determine a flight plan that ensures recognition of all major targets.

The article discussed an algorithm used for detailed flight route planning, which takes into account the payload installed on the UAV. The optimization algorithm was presented, used to determine the exact flight trajectory. Detailed algorithm for determining the turns in flight was presented also. The latter algorithm belongs to the group of algorithms for plotting a trajectory based on Dubin curves, but it is very easy to implement, unlike many others presented in the literature. The set of algorithms presented allows for the implementation of the flight routing subsystem for one or several UAVs that use payload for target recognition. Therefore, the article contains a complete set of algorithms that allow detailed mission planning on the air platform using available commercial or non-commercial solvers.

Further work is associated with the implementation of methods for dynamic change of a UAV flight plan, which is a difficult task because tactical class systems are not equipped with appropriate number of sensors. In addition, it should be noted that UAV that performs reconnaissance tasks, is not usually integrated with other sources of reconnaissance data. In terms of optimization, especially the graph and network theory, to perform this task, the presented model should be extended with mechanisms for efficient modification of the connection network, i.e., the construction of the so-called ad hoc networks.

The need to expand the presented model with elements of network modification in the ad hoc mode forces the use of video tracking mechanisms in practical operational activities. In this case, the algorithms of the EO/IR alone are insufficient (for SAR, such algorithms are not yet implemented, although researches are ongoing) and the mechanisms of flight route planning should be modified due to the mutual positioning of the UAV and the tracked target.

Considering the dynamics of changes in the battlefield situation and the fact that tactical class UAVs are the outermost operational element, it will be required to increase their recognition capabilities through the use of ELINT electronic reconnaissance subsystems on board UAVs.

It is also worth emphasizing the important issue mentioned in the paper. The issue involves the need to build models for specific classes of unmanned systems. Each optimization model presented in the paper for solving the route planning task and determining flight trajectories concerned a tactical class system. Detachment from the UAV class and its equipment is too much simplification of the problem and usually makes models purely theoretical.

It is only when the flight trajectories are planned in detail for the UAV mission that it is possible to start further tasks related to the location of the unmanned platform in the field in the event of loss of GPS signal. It is currently one of the most strongly developed directions of work in the field of building intelligent flying systems.
